# Supplementary material for: Unveiling the Bioactive Potential of the Invasive Jellyfish Phyllorhiza punctata Through Integrative Transcriptomic and Proteomic Analyses
Source: Biomolecules. 2025 Aug 4;15(8):1121. doi: 10.3390/biom15081121 (PMC12383608; doi:10.3390/biom15081121)
Supplement: Supplementary file 1 [file biomolecules-15-01121-s001.zip › Suplementary Material/FileS3/PEPFOLD4/XP_065062330_9809/clusters.html]

 PEP-FOLD clusters


Download archive of all models  here   

|  |  |  |
| --- | --- | --- |
| # | file | sOPEP |
| # cluster 1 - Size: 32 |  |  |
|  | XP\_065062330\_9809-model1 | -26.5297 |
|  | XP\_065062330\_9809-model1.1 | -26.4275 |
|  | XP\_065062330\_9809-model1.2 | -26.1823 |
|  | XP\_065062330\_9809-model1.3 | -26.0509 |
|  | XP\_065062330\_9809-model1.4 | -25.9691 |
|  | XP\_065062330\_9809-model1.5 | -25.5266 |
|  | XP\_065062330\_9809-model1.6 | -25.451 |
|  | XP\_065062330\_9809-model1.7 | -25.4375 |
|  | XP\_065062330\_9809-model1.8 | -25.4308 |
|  | XP\_065062330\_9809-model1.9 | -25.3224 |
|  | XP\_065062330\_9809-model1.10 | -25.1481 |
|  | XP\_065062330\_9809-model1.11 | -25.1404 |
|  | XP\_065062330\_9809-model1.12 | -25.1103 |
|  | XP\_065062330\_9809-model1.13 | -24.9611 |
|  | XP\_065062330\_9809-model1.14 | -24.9321 |
|  | XP\_065062330\_9809-model1.15 | -24.8587 |
|  | XP\_065062330\_9809-model1.16 | -24.8472 |
|  | XP\_065062330\_9809-model1.17 | -24.8277 |
|  | XP\_065062330\_9809-model1.18 | -24.8167 |
|  | XP\_065062330\_9809-model1.19 | -24.6603 |
|  | XP\_065062330\_9809-model1.20 | -24.6381 |
|  | XP\_065062330\_9809-model1.21 | -24.5511 |
|  | XP\_065062330\_9809-model1.22 | -24.5444 |
|  | XP\_065062330\_9809-model1.23 | -24.5408 |
|  | XP\_065062330\_9809-model1.24 | -24.5365 |
|  | XP\_065062330\_9809-model1.25 | -24.5355 |
|  | XP\_065062330\_9809-model1.26 | -24.5073 |
|  | XP\_065062330\_9809-model1.27 | -24.4087 |
|  | XP\_065062330\_9809-model1.28 | -24.3405 |
|  | XP\_065062330\_9809-model1.29 | -24.2678 |
|  | XP\_065062330\_9809-model1.30 | -24.224 |
|  | XP\_065062330\_9809-model1.31 | -24.2142 |
| # cluster 2 - Size: 4 |  |  |
|  | XP\_065062330\_9809-model2 | -26.4548 |
|  | XP\_065062330\_9809-model2.1 | -26.2789 |
|  | XP\_065062330\_9809-model2.2 | -25.9939 |
|  | XP\_065062330\_9809-model2.3 | -25.8418 |
| # cluster 3 - Size: 23 |  |  |
|  | XP\_065062330\_9809-model3 | -26.0721 |
|  | XP\_065062330\_9809-model3.1 | -25.3873 |
|  | XP\_065062330\_9809-model3.2 | -25.2063 |
|  | XP\_065062330\_9809-model3.3 | -25.1578 |
|  | XP\_065062330\_9809-model3.4 | -25.0201 |
|  | XP\_065062330\_9809-model3.5 | -25.006 |
|  | XP\_065062330\_9809-model3.6 | -24.8758 |
|  | XP\_065062330\_9809-model3.7 | -24.8751 |
|  | XP\_065062330\_9809-model3.8 | -24.7898 |
|  | XP\_065062330\_9809-model3.9 | -24.7513 |
|  | XP\_065062330\_9809-model3.10 | -24.7368 |
|  | XP\_065062330\_9809-model3.11 | -24.7308 |
|  | XP\_065062330\_9809-model3.12 | -24.6907 |
|  | XP\_065062330\_9809-model3.13 | -24.4592 |
|  | XP\_065062330\_9809-model3.14 | -24.3697 |
|  | XP\_065062330\_9809-model3.15 | -24.2712 |
|  | XP\_065062330\_9809-model3.16 | -24.2493 |
|  | XP\_065062330\_9809-model3.17 | -24.2408 |
|  | XP\_065062330\_9809-model3.18 | -24.2344 |
|  | XP\_065062330\_9809-model3.19 | -24.0134 |
|  | XP\_065062330\_9809-model3.20 | -23.9885 |
|  | XP\_065062330\_9809-model3.21 | -23.9252 |
|  | XP\_065062330\_9809-model3.22 | -23.8923 |
| # cluster 4 - Size: 1 |  |  |
|  | XP\_065062330\_9809-model4 | -25.7696 |
| # cluster 5 - Size: 13 |  |  |
|  | XP\_065062330\_9809-model5 | -25.6469 |
|  | XP\_065062330\_9809-model5.1 | -25.1017 |
|  | XP\_065062330\_9809-model5.2 | -25.0771 |
|  | XP\_065062330\_9809-model5.3 | -24.8066 |
|  | XP\_065062330\_9809-model5.4 | -24.6511 |
|  | XP\_065062330\_9809-model5.5 | -24.6102 |
|  | XP\_065062330\_9809-model5.6 | -24.4238 |
|  | XP\_065062330\_9809-model5.7 | -24.3812 |
|  | XP\_065062330\_9809-model5.8 | -24.2395 |
|  | XP\_065062330\_9809-model5.9 | -24.2311 |
|  | XP\_065062330\_9809-model5.10 | -24.217 |
|  | XP\_065062330\_9809-model5.11 | -24.1056 |
|  | XP\_065062330\_9809-model5.12 | -24.0065 |
| # cluster 6 - Size: 25 |  |  |
|  | XP\_065062330\_9809-model6 | -24.8479 |
|  | XP\_065062330\_9809-model6.1 | -24.8177 |
|  | XP\_065062330\_9809-model6.2 | -24.7373 |
|  | XP\_065062330\_9809-model6.3 | -24.7274 |
|  | XP\_065062330\_9809-model6.4 | -24.7228 |
|  | XP\_065062330\_9809-model6.5 | -24.7043 |
|  | XP\_065062330\_9809-model6.6 | -24.6133 |
|  | XP\_065062330\_9809-model6.7 | -24.6082 |
|  | XP\_065062330\_9809-model6.8 | -24.5859 |
|  | XP\_065062330\_9809-model6.9 | -24.5644 |
|  | XP\_065062330\_9809-model6.10 | -24.54 |
|  | XP\_065062330\_9809-model6.11 | -24.5267 |
|  | XP\_065062330\_9809-model6.12 | -24.5188 |
|  | XP\_065062330\_9809-model6.13 | -24.4563 |
|  | XP\_065062330\_9809-model6.14 | -24.4387 |
|  | XP\_065062330\_9809-model6.15 | -24.4168 |
|  | XP\_065062330\_9809-model6.16 | -24.3196 |
|  | XP\_065062330\_9809-model6.17 | -24.2028 |
|  | XP\_065062330\_9809-model6.18 | -24.1952 |
|  | XP\_065062330\_9809-model6.19 | -24.1878 |
|  | XP\_065062330\_9809-model6.20 | -24.1663 |
|  | XP\_065062330\_9809-model6.21 | -24.1489 |
|  | XP\_065062330\_9809-model6.22 | -24.1012 |
|  | XP\_065062330\_9809-model6.23 | -24.063 |
|  | XP\_065062330\_9809-model6.24 | -23.947 |
| # cluster 7 - Size: 2 |  |  |
|  | XP\_065062330\_9809-model7 | -24.6436 |
|  | XP\_065062330\_9809-model7.1 | -24.3232 |
|  |  |  |
